# Langerhans cell histiocytosis at L5 vertebra treated with en bloc vertebral resection: a case report

**DOI:** 10.1186/s12957-018-1399-1

**Published:** 2018-05-22

**Authors:** Lunhao Chen, Zhong Chen, Yue Wang

**Affiliations:** Spine Lab, Department of Orthopedic Surgery, The First Affiliated Hospital, School of Medicine, Zhejiang University, The second floor of Building 3, 79# Qingchun Road, Hangzhou, 310003 China

**Keywords:** Langerhans cell histiocytosis, En bloc, Vertebral resection, Lumbar spine, Surgery

## Abstract

**Background:**

Langerhans cell histiocytosis (LCH) in adult lumbar spine is extremely rare, and optimal treatments remain unclear. In literature, only a few cases of lumbar spine LCH were treated using surgery but en bloc vertebral resection has not been used.

**Case presentation:**

A 50-year-old man presented with unbearable radiating pain at his right leg. Radiological studies revealed a solitary osteolytic lesion, which was moderately enhanced on contrast MR imaging and hyper-metabolic on PET/CT, at the right L5 vertebral body and arch. In biopsy, Langerhans cells were observed, but findings were insufficient to establish a diagnosis of LCH. A modified L5 en bloc vertebral resection via anterior and posterior approaches was performed to remove the right 2/3 portion of the L5 vertebra. The left 1/3 vertebral body and left pedicle of L5, which were not affected, were kept in situ to allow short instrumentation and reconstruction. His leg pain disappeared after the surgery, and a precise diagnosis of LCH was established after a throughout histological study of the removed vertebra. The patient further accepted 1 cycle of low-dose radiotherapy postoperatively. At 18-month follow-up, the lumbosacral spine was fused and no local reoccurrence was noticed.

**Conclusions:**

For lumbar spine LCH, surgery should be considered if there are neurological symptoms or histological diagnosis is indefinite in biopsy. En bloc vertebral resection can be used to alleviate neurological symptoms and prevent local reoccurrence.

## Background

Langerhans cell histiocytosis (LCH) is a proliferative disease that origins from dendritic cell family [1]. LCH is rare, with an estimated point prevalence of 1 case in 1.5 million [2]. LCH often involve multiple systems, and clinical presentations vary considerably [3, 4]. Osseous lesions are the most common findings in LCH, with approximately 60% of LCH patients had one or more lytic osseous lesions [3]. Typically, skull and femur are the most common bones involved. In rare case, osseous lesions occur in the spine, resulting in back pain, radiculopathy, or neurological deficits [5]. For spinal LCH, a standard treatment protocol is currently absent. Here, we report a rare case of LCH at the L5 vertebra which was treated with en bloc vertebral resection.

## Case presentation

An otherwise healthy 50-year-old gentleman consulted us for his radiating pain at the right leg. The pain, which was triggered by 10-h driving 1 month ago, started from the right buttock and radiated down to the sole of the foot. The leg pain was rather severe, intolerable sometimes, and he cannot stand or walk when the pain attacked. He sought for treatment at a local hospital, and magnetic resonance (MR) imaging revealed an osseous lesion in the L5 vertebra. He was suspected to have lumbar metastasis and was referred to us for further treatment. He had no fever, no weight loss, and no bowel or bladder problems since he had the pain.

On physical examinations, he rated the leg pain as visual analogue scale (VAS) 7 points. His right straight leg raising test was positive. There was haphalgesia on his right calf, but no obvious sensation loss in the right leg. Manual muscle tests (MMT) revealed slightly decreased muscle power in his right tibialis anterior, gastrocnemius, and ankle dorsiflexors (grade IV^+^). His bilateral knee and ankle reflexes were normal. Pathological reflexes were negative for both legs.

**Fig. 1:**
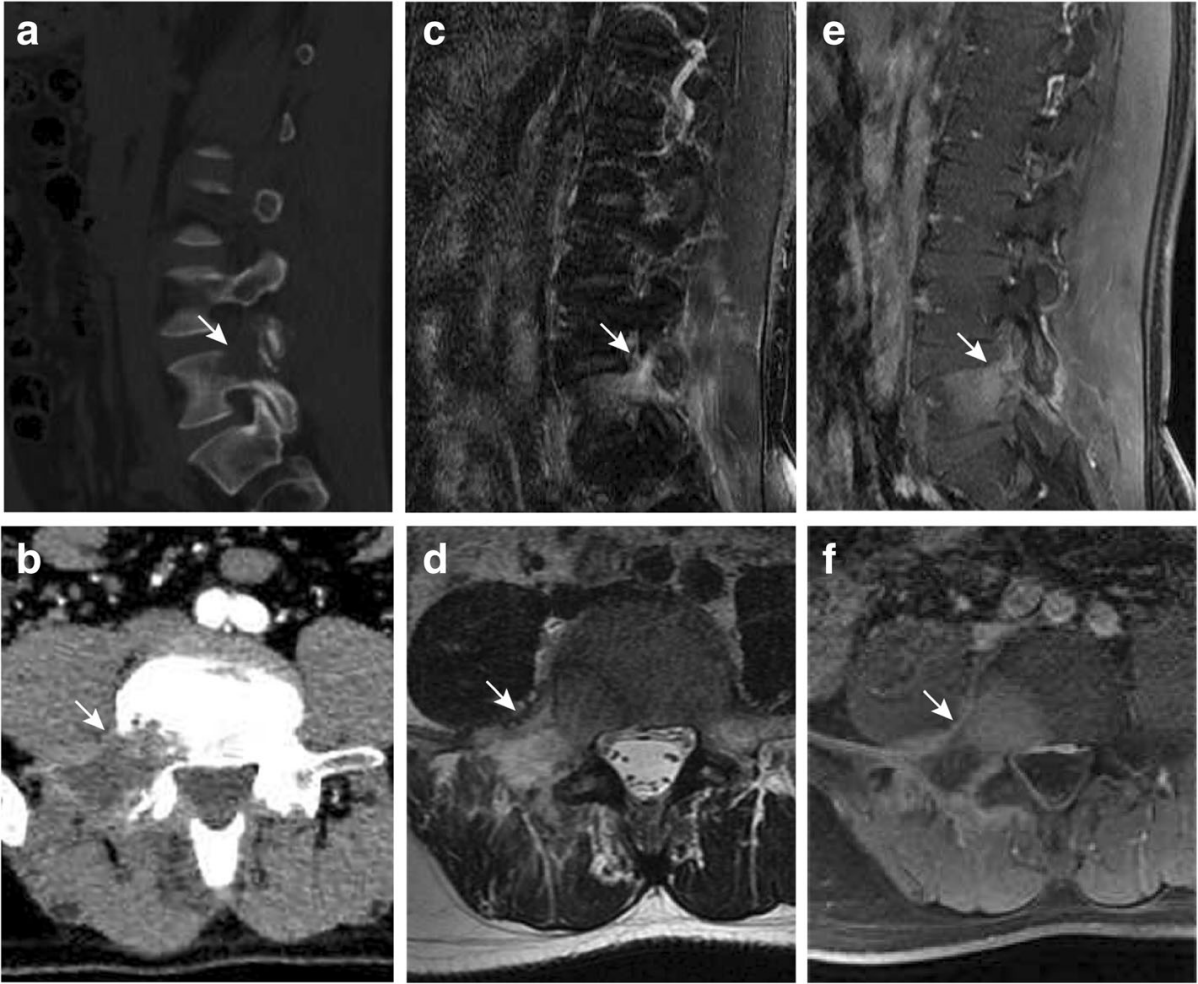
Radiological findings in the lumbar spine. **a** Sagittal CT demonstrated a lytic lesion involved in the vertebral body, right pedicle, and transverse process. **b** Contrast CT revealed a mostly non-enhanced vertebral lesion. The lesion is hyper-intense on T2W MR images (**c**, **d**) and moderately enhanced on gadolinium contrast MR images (**e**, **f**). Paravertebral soft tissues surrounding also seems to be involved

**Fig. 2:**
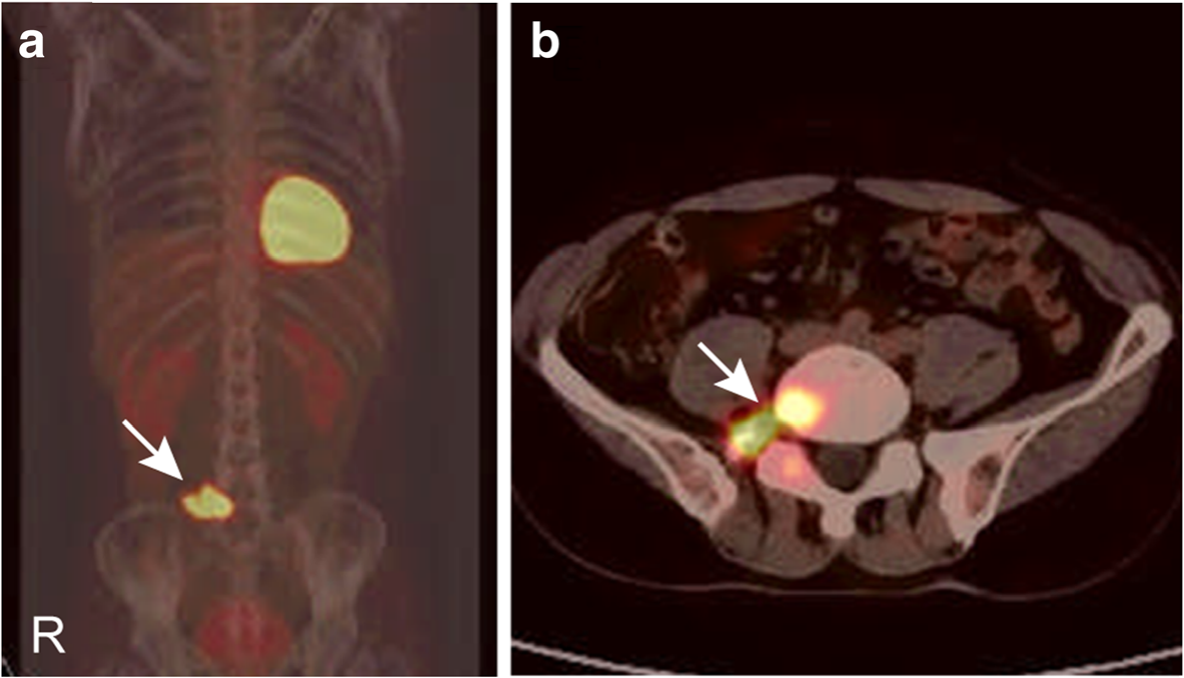
PET/CT showed a single site of increased intake of ^18^F-FDG in the right L5 vertebra (**a**, **b**). The maximal standardized uptake value (SUV) was 10.7 for L5 vertebra and 1.6 for his liver, which was suggestive of a malignant tumor

On computed tomography (CT) images, the right portion of the L5 vertebra, including the vertebral body, pedicle, and transverse process, was destructed (Fig. 1a). Contrast CT revealed a mostly non-enhanced lytic lesion at the right L5 vertebral arch and body (Fig. 1b). The lesion, which was hypo-intense on T1-weighted (T1W) and hyper-intense on T2-weighted (T2W) sequences, mainly involved the right portion of vertebral body and arch and was moderately enhanced on gadolinium contrast sequence (Fig. 1c–f). Positron emission tomography/computed tomography (PET/CT) revealed a single site of increased uptake of ^18^F-fluorodeoxyglucose (^18^F-FDG) at the right portion of the L5 vertebra (Fig. 2).

The patient underwent percutaneous needle biopsy. Histological studies reported the presence of some Langerhans cells in the tissue sample. Yet, histological evidence was not sufficient to make a conclusive diagnosis of LCH, and spine metastasis cannot be completely excluded due to some atypical cellular characteristics. The surgical team discussed with the patient and his family further treatment options, including repeated percutaneous biopsy, open biopsy, surgical curettage, vertebral resection, radiation therapy, chemotherapy, and some other possible interventions. After comprehensive discussions, the patient selected surgical removal of the vertebral lesion.

**Fig. 3:**
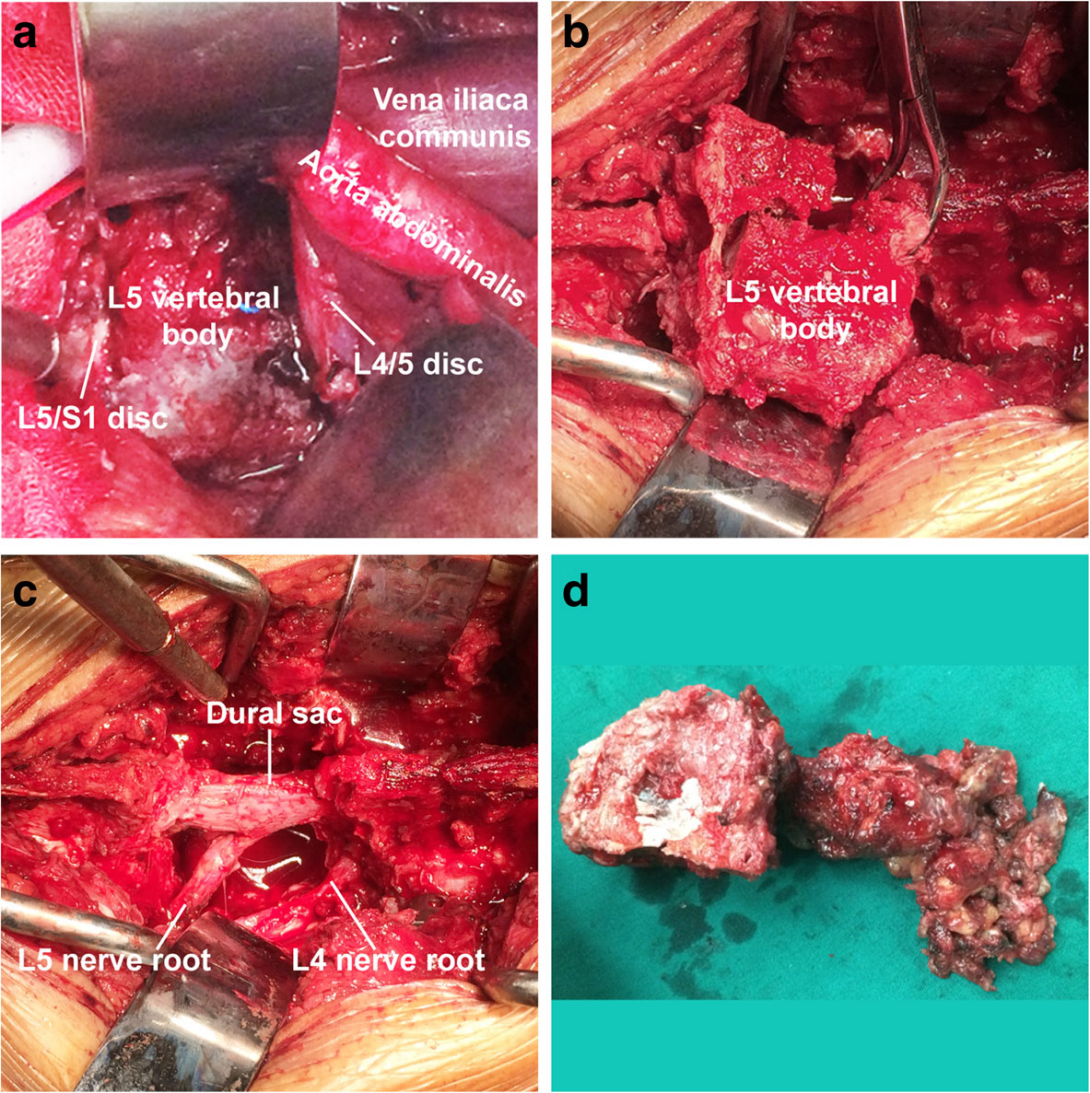
A modified L5 en bloc vertebral resection was performed using anterior and posterior approaches. **a** The anterior portions of L4–S1 vertebrae were exposed using an anterior approach. **b** The L5 vertebra was rotated out through the posterior approach. **c** The vertebra was removed, leaving the nerve roots and dural sac intact. **d** A gross view of the removed vertebra and lesion

**Fig. 4:**
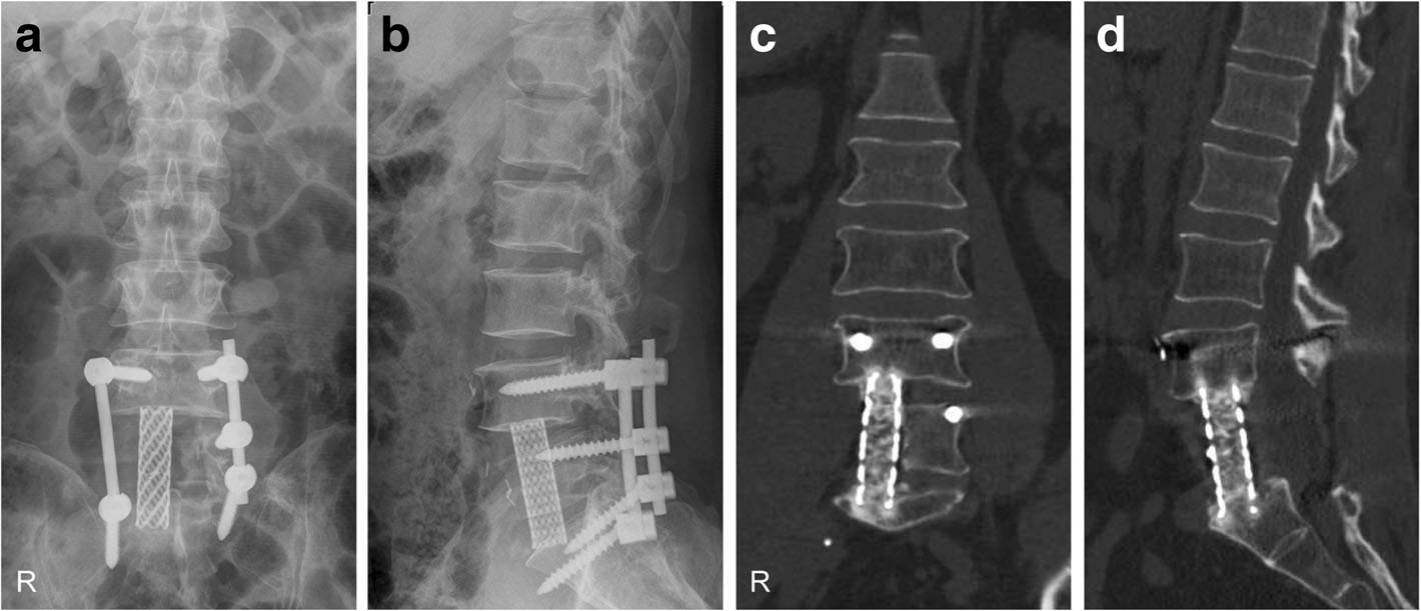
Radiographs of the lumbosacral spine 1 week after surgery (**a**, **b**). At 18-month follow-up, CT images demonstrated that L4–S1 vertebrae were fused through the mesh (**c**, **d**)

A modified L5 en bloc vertebral resection via anterior and posterior approaches was designed. As the lesion merely involved the right portion of the L5 vertebra, the left 1/3 portion were kept in situ to enhance segmental stability for the use of short instrumentation. First, a transabdominal approach was used to detach the right psoas and soft tissues from the L5 vertebra (Fig. 3a), remove the right portion of L4/5 and L5/S1 intervertebral discs, and sagittally split the L5 vertebral body at the junction between right 2/3 and left 1/3. Then, a posterior approach was used to perform laminectomy, and remove the disassociated L5 vertebral body **(**Fig. 3b, c), pedicle screw (L4, S1, and the left L5 pedicle), and cage (with autologous lamina bone graft) instrumentation (Fig. 4a, b). There was 1500 ml blood loss in the surgery.

**Fig. 5:**
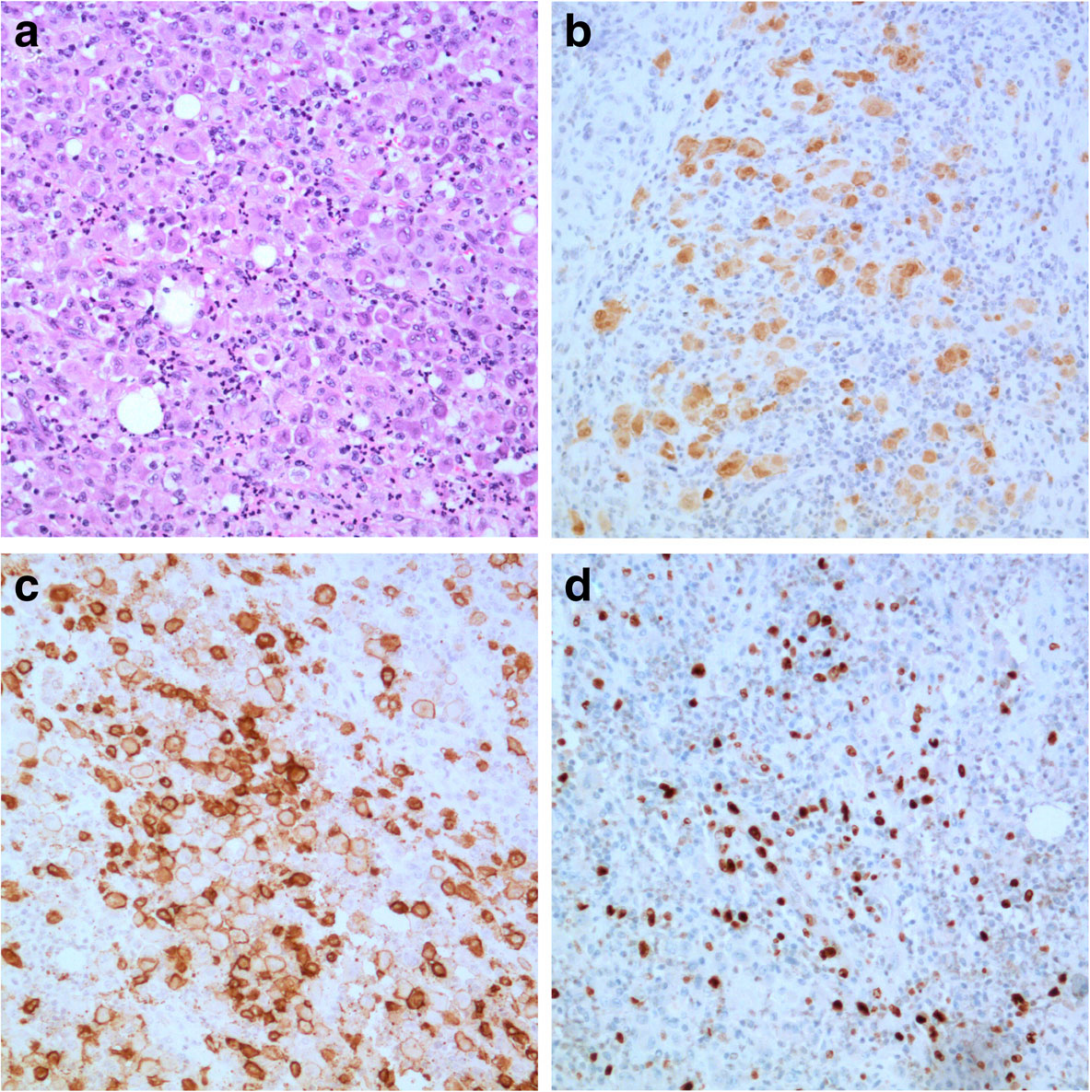
Histological study of the removed vertebra. HE staining (**a**) demonstrated numerous eosinophils cells in the tissue. S-100β (**b**) and CD-1a (**c**) immunochemistry staining revealed typical Langerhans cells. Immunochemistry staining for Ki-67 (**d**) showed cells were under active proliferation

The patient’s leg pain disappeared immediately after surgery. There was a generally decreased muscle power in his right leg (MMT grade IV) and consistent numbness in his right calf and foot. These symptoms, however, did not impair his mobility. With a brace, the patient walked independently 2 weeks after the surgery. A throughout histological study of the removed vertebra revealed numerous typical Langerhans cells, which were positive for S-100β and CD-1a (Fig. 5b, c). Ki-67 immunochemistry staining demonstrated that the Langerhans cells are active in proliferation (Fig. 5d). A pathological diagnosis of LCH was soundly established. The patient further accepted 1 cycle of low-dose radiotherapy (7 days) to eliminate potential remaining lesions in the surgical site. Mecobalamine was given for 12 months during his rehabilitation.

At 18-month follow-up, the patient had minor numbness in his ankle. His right calf was a bit thinner than the left, but no power weakness was noticed. He has returned to previous work and normal life 1 year ago. CT scan of the lumbar spine revealed that L4–S1 vertebrae were fused through the titanium mesh (Fig. 4c, d). No sign of reoccurrence was noticed in the lumbar spine.

## Discussion

The current case highlighted a rare case of LCH in the L5 vertebra with nerve root compression which was treated with extensive surgical excision. Reportedly, only 4.1% of LCH osseous lesions occur in the lumbar spine [3], and involvement of spinal canal, nerve root or, paravertebral tissue is extremely rare [6]. In literature, no more than 10 cases of lumbar LCH were reported [5, 7–12], and four of them were treated with partial or complete surgical excision [8, 9, 11, 12]. In the present case, the intolerable neurological pain, indefinite diagnosis, and suspicious vertebral metastasis reported in biopsy led to a radical surgery to remove the lesion. Yet, the involvement of anterior and posterior portions of the L5 vertebra complicates the surgery. A modified en bloc vertebral resection via anterior and posterior approaches was performed, and the lumbosacral spine was reconstructed using short instrumentation. The patient had a favorable clinical outcome in short-term follow-up.

Routine radiological studies, such as X-ray, CT, and MR imaging, are able to identify osseous lesions but have little value in differentiating LCH from other osteolytic tumors. LCH typically exhibits hypo-intensity on T1W and hyper-intensity on T2W images and moderately enhancement on contrast MR images [12]. PET/CT is sensitive to detect hyper-metabolic lesions and multiple involvements of LCH. Specificities of these radiological approaches are low for the diagnosis of LCH, and none of them can lead to a definite diagnosis of LCH.

A precise diagnosis of LCH largely relies on biopsy and histological study [4]. Using percutaneous needle biopsy guided by CT or C-arm, reportedly, a definitive diagnosis can be established in 82–90% of cases of LCH [13, 14]. Still, there are a considerable number of cases that cannot be precisely diagnosed in needle biopsy. This may be due to either inadequate tissue sample obtained in needle biopsy or lack of typical cellular findings in histological study [14]. As in the current case, percutaneous needle biopsy failed to establish a conclusive diagnosis of LCH, though Langerhans cells were observed. According to *World Health Organization classification of tumors of soft tissue and bone* [15], LCH belongs to tumors of undefined neoplastic nature which may share some biological characteristics, such as osteolytic lesion, exophytic growth, and even distant metastasis, with some other bone neoplasms [3]. Moreover, it is noteworthy that LCH and other neoplasms sometimes may co-exist in a patient or even within a lesion [16, 17]. Approximately 4% of patients with LCH had concomitant neoplasms diagnosed at or after the time a diagnosis of LCH was established [3].

While there is no consensus on the management of LCH, a number of approaches, including surgery, chemotherapy, radiotherapy, glucocorticoid, monoclonal antibody, and even nonsteroidal anti-inflammatory drugs (NSAIDS), were used to treat LCH [5, 11, 18, 19]. For skeletal LCH, surgery should be considered if there is neurological involvement or histological diagnosis was not confident in biopsy [20–22]. Surgical excision is an effective treatment for LCH, especially in patients with solitary bone lesions or neurological symptoms [3, 9]. In a case series of LCH in adults, 75% of who received marginal removal and 83% of who received simple curettage had no signs of local recurrence in an average of 8.5-year follow-up, which was better than those treated with chemotherapy or steroids (about 50% non-reoccurrence rate) [18].

Currently, the optimal treatments for spinal LCH remain controversial. While most cases of spinal LCH were treated conservatively, surgical curettage or excision was also reported [6, 9, 11]. Occasionally, solidary lumbar LCH was treated with hemi-vertebral resection through a posterolateral approach [9]. En bloc vertebral resection is a developed surgery which has been proven to be efficacious in treating solitary spine tumors [23, 24], and particularly can reduce the chance of local recurrence [25]. To our knowledge, this is the first time that en bloc technique was used to treat spinal LCH. In this special case, instead of a traditional en bloc vertebral resection, we kept the left 1/3 L5 vertebra to reduce surgical time, maintain segmental stability, and shorten spinal instrumentation. As is the case, the patient was mobile shortly after the surgery, and solid lumbosacral fusion was achieved on time.

One may doubt if such a radical surgery is too aggressive for the current patient. It is possible that the patient can be managed using chemotherapy or radiotherapy, if a precise diagnosis of LCH can be established in biopsy. Yet, it is not clear if symptoms of nerve root compression can be relieved using non-surgical treatments. While surgery can quickly alleviate pain symptoms, radical removal may be better than local excision to achieve disease-free survival.

## Conclusions

We reported a rare case of adult LCH in the L5 vertebra with neurological compression. The patient was successfully treated using a modified en bloc vertebral resection. Although aggressive, en bloc vertebral resection was effective to quickly relieve pain symptoms and prevent local reoccurrence in treating spinal LCH.
